# Supplementary material for: Zinc-Mediated Template Synthesis of Hierarchical Porous N-Doped Carbon Electrocatalysts for Efficient Oxygen Reduction
Source: Molecules. 2023 May 23;28(11):4257. doi: 10.3390/molecules28114257 (PMC10254328; doi:10.3390/molecules28114257)
Supplement: Supplementary file 1 [file molecules-28-04257-s001.zip › molecules-2372940-supplementary.pdf]

Supplementary Materials

Zinc-Mediated Template Synthesis of Hierarchical Porous N-Doped Carbon Electrocatalysts for Efficient Oxygen Reduction

Qianhui Ma [1,†], Guifa Long [2,†], Xulei Tang [1], Xiaobao Li [1], Xianghui Wang [1,*], Chenghang You [3], Wenjun Fan [4], Qingqing Wang [1,3,*]

[1] School of Chemistry and Chemical Engineering, Hainan Normal University, Haikou 571158, China

[2] Guangxi Key Laboratory of Chemistry and Engineering of Forest Products, School of Chemistry and Chemical Engineering, Guangxi Minzu University, Nanning 530008, China

[3] State Key Laboratory of Marine Resource Utilization in South China Sea, Hainan Provincial Key Lab of Fine Chemistry, School of Chemical Engineering and Technology, Hainan University, Haikou 570228, China

[4] Dalian National Laboratory for Clean Energy, State Key Laboratory of Catalysis, iChEM, Dalian Institute of Chemical Physics, Chinese Academy of Sciences, Dalian 116023, China

* Correspondence: god820403@163.com (X.W.); 001qinger@163.com (Q.W.)

† These authors contributed equally.

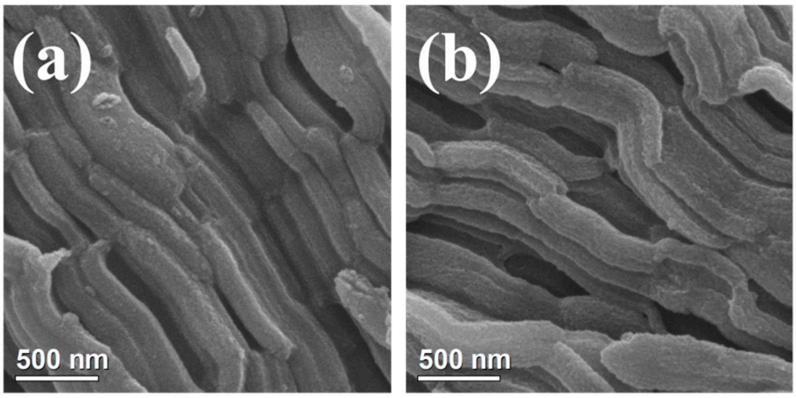

Figure S1 SEM images: (a) NC-0.4-S; (b) NC-0.6-S

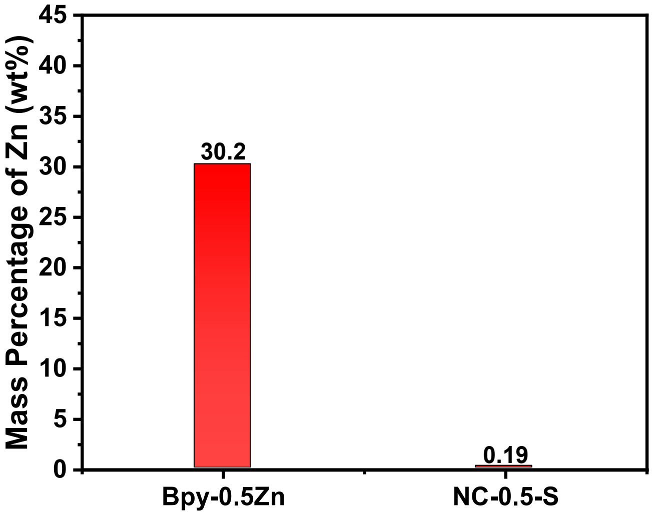

Figure S2 ICP-AES results for Bpy-0.5Zn and NC-0.5-S

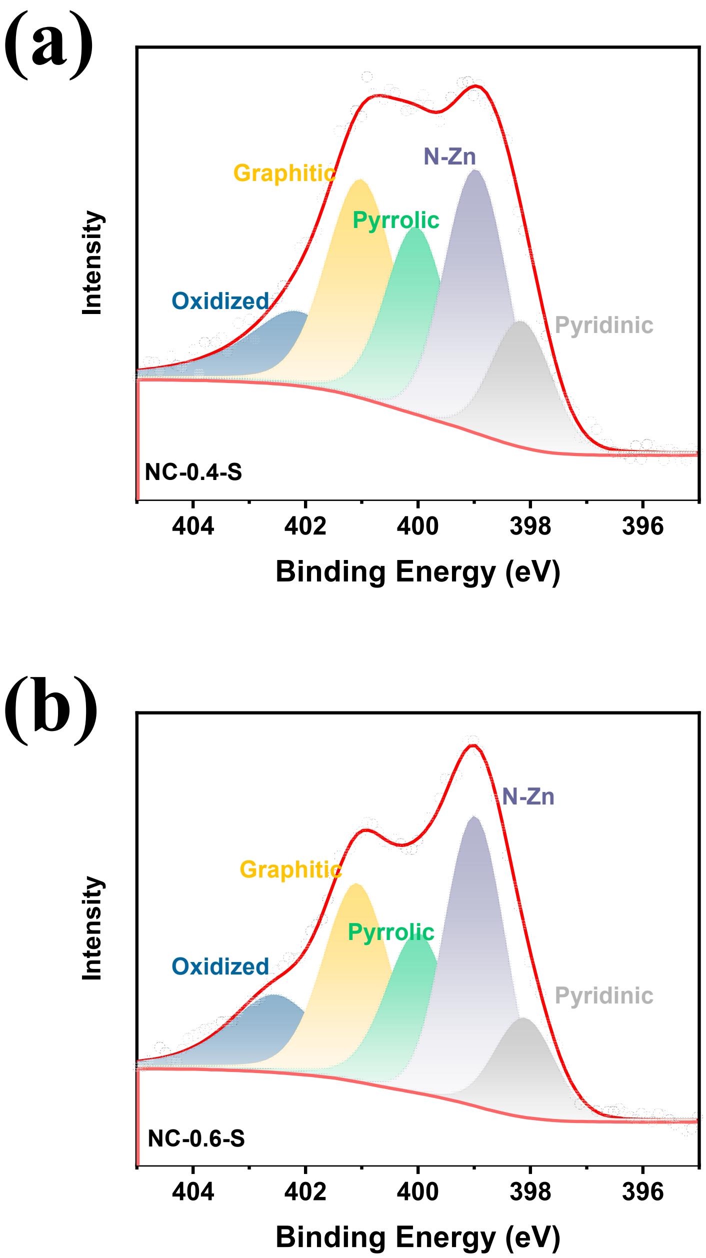

Figure S3 N1s spectra: (a) NC-0.4-S; (b) NC-0.6-S

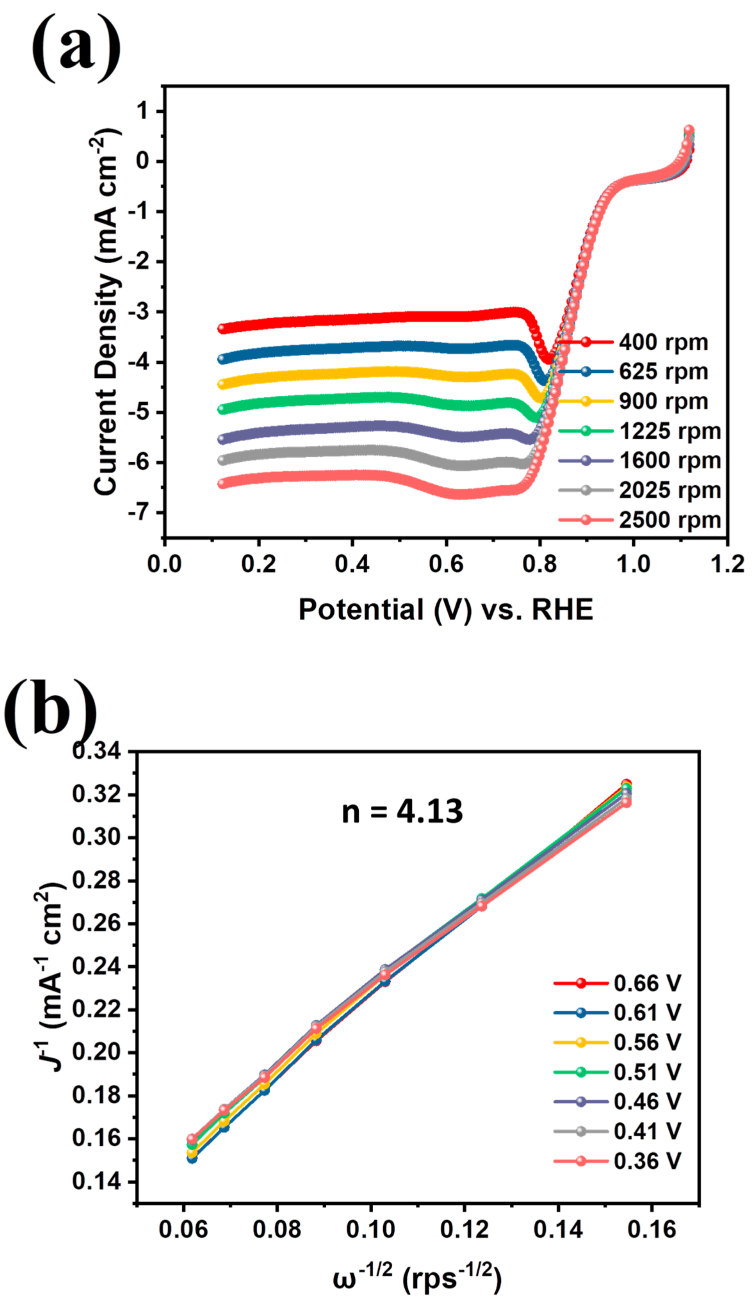

Figure S4 (a) LSV curves under different rotating rates of NC-0.5-S; (b) Koutecky-Levich (K-L) plots and calculated electron transfer number (n).

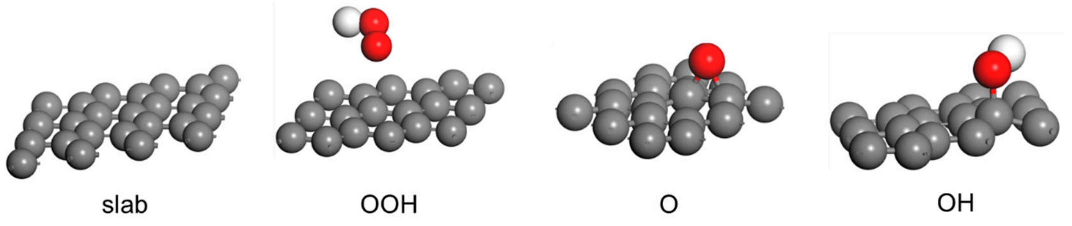

Figure S5 Optimized structures of C1 and the intermediate species adsorbed on it.

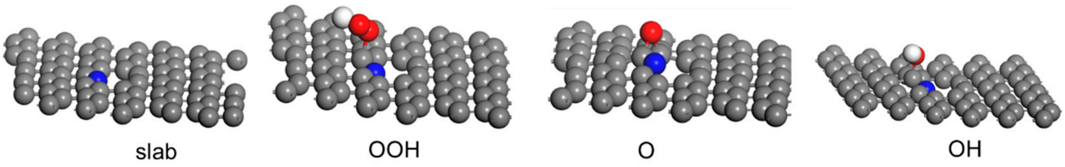

Figure S6 Optimized structures of C2 and the intermediate species adsorbed on it.

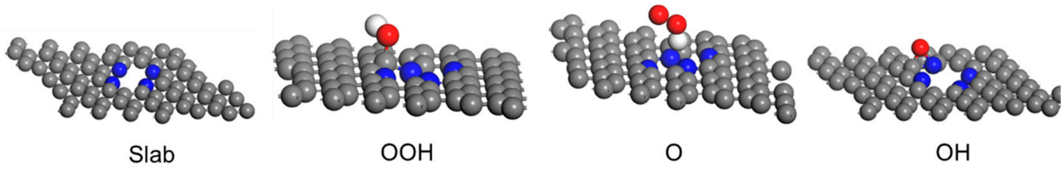

Figure S7 Optimized structures of C3 and the intermediate species adsorbed on it.

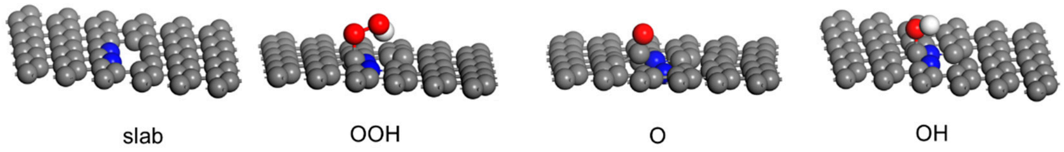

Figure S8 Optimized structures of C4 and the intermediate species adsorbed on it.

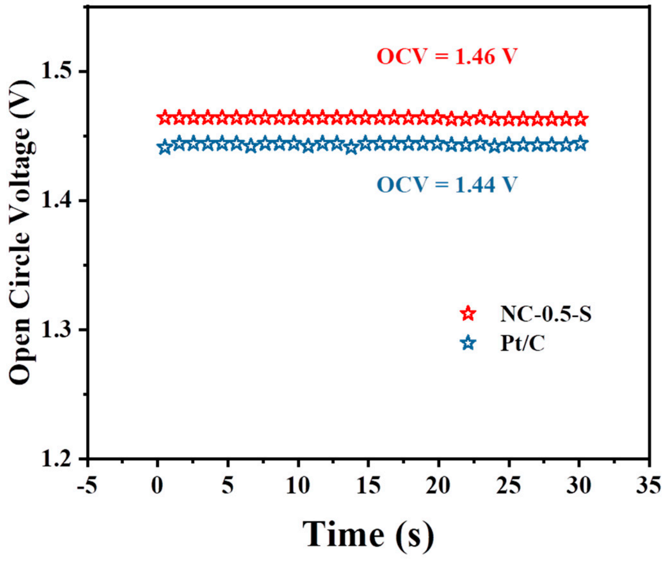

Figure S9 The open circle potentials for the ZABs using NC-0.5-S and Pt/C

Table S1 surface atomic compositions of various catalysts derived from the XPS results

	Bpy	Bpy-0.5Zn	NC-0.5	NC-0-S	NC-0.4-S	NC-0.5-S	NC-0.6-S
**C1s**	78.77	57.64	89.98	85.02	88.61	89.32	87.21
**N1s**	8.42	9.59	6.17	4.82	6.62	7.14	7.95
**O1s**	12.81	27.98	3.48	10.16	4.6	3.42	4.73
**Zn2p**	-	4.79	0.37	-	0.16	0.12	0.11

Table S2 The adsorption energies of intermediate species of ORR on different C sites.

	C1	C2	C3	C4
ΔG_{*OOH} / eV	5.31	4.78	4.69	1.0
ΔG_{*O} / eV	3.51	3.38	3.66	-2.51
ΔG_{*OH} / eV	3.07	2.81	2.57	-1.97

Table S3 comparison of NC-0.5-S and other catalysts for the zinc-air battery recently reported

No.	Catalyst	Half-wave potential (V vs. RHE)	Peak power density (mW cm^{-2})	Specific capacity (mAhg_{Zn}^{-1})	Reference
1	CoSA/N,S-HCS	0.85	1.5	173.1	[1]
2	FeCoMoS@NG	0.83	1.44	118	[2]
3	CuSA@HNCNx	0.91	1.51	212	[3]
4	CS-NFO@PNC-700	0.85	1.45	130	[4]
5	LDH-POF	0.8		185	[5]
6	VO-CMON@NCN	0.857	1.404	143.7	[6]
7	S-CFZ	0.82		178	[7]
8	CMO–U@CC	0.81	1.42	135	[8]
9	Vo-CoFe/CoFe2O4@NC	0.858	1.53	139.5	[9]
10	Co/ZnCo2O4@NC-CNT	0.9	1.47	305	[10]
11	NiFe/bNCNT	0.8	1.52	224	[11]
12	Co/CoS/Fe-HSNC	0.9		213	[12]
13	CoDNG900	0.864	1.45	205	[13]
14	FeNi–SAs@NC	0.907	1.54	260	[14]
15	Co3O4/Co@NCs	0.92	1.5	123.4	[15]
16	CoFe–Co@PNC	0.887	1.46	152.8	[16]
17	Co/N@CNTs@CNMF	0.86	1.52	133	[17]
18	Fe,Mn/N–C	0.928	1.4	160.8	[18]
19	FeNi/N-CPCF-950	0.89	1.478	160.6	[19]
20	BFC-FC-0.2	0.9	1.49	160	[20]
21	CoNi/Co–N@HNC	0.86	1.43	179.1	[21]
22	CoFe/Co@NCNT/NG	0.876	1.4	161	[22]
23	FeNiN-MWCNT	0.87	1.45	84.5	[23]
	NC-0.5-S	0.89	1.46	196.3	**Our work**

References

1. Zhang, Z.; Zhao, X.; Xi, S.; Zhang, L.; Chen, Z.; Zeng, Z.; Huang, M.; Yang, H.; Liu, B.; Pennycook, S. J., Atomically dispersed cobalt trifunctional electrocatalysts with tailored coordination environment for flexible rechargeable Zn–air battery and self‐driven water splitting. *Adv. Energy Mater.* **2020,** 10, (48), 2002896.
2. Ramakrishnan, S.; Balamurugan, J.; Vinothkannan, M.; Kim, A. R.; Sengodan, S.; Yoo, D. J., Nitrogen-doped graphene encapsulated FeCoMoS nanoparticles as advanced trifunctional catalyst for water splitting devices and zinc–air batteries. *Appl. Catal., B* **2020,** 279, 119381.
3. Wagh, N. K.; Shinde, S. S.; Lee, C. H.; Jung, J.-Y.; Kim, D.-H.; Kim, S.-H.; Lin, C.; Lee, S. U.; Lee, J.-H., Densely colonized isolated Cu-N single sites for efficient bifunctional electrocatalysts and rechargeable advanced Zn-air batteries. *Appl. Catal., B* **2020,** 268, 118746.
4. Ramakrishnan, S.; Velusamy, D. B.; Sengodan, S.; Nagaraju, G.; Kim, D. H.; Kim, A. R.; Yoo, D. J., Rational design of multifunctional electrocatalyst: An approach towards efficient overall water splitting and rechargeable flexible solid-state zinc–air battery. *Appl. Catal., B* **2022,** 300, 120752.
5. Zhao, C. X.; Liu, J. N.; Li, B. Q.; Ren, D.; Chen, X.; Yu, J.; Zhang, Q., Multiscale Construction of Bifunctional Electrocatalysts for Long‐Lifespan Rechargeable Zinc–Air Batteries. *Adv. Funct. Mater.* **2020,** 30, (36), 2003619.
6. Nguyen, T. T.; Balamurugan, J.; Lau, K.-T.; Kim, N. H.; Lee, J. H., Novel cobalt-doped molybdenum oxynitride quantum dot@N-doped carbon nanosheets with abundant oxygen vacancies for long-life rechargeable zinc–air batteries. *J. Mater. Chem. A* **2021,** 9, (14), 9092-9104.
7. Jiang, Y.; Deng, Y.-P.; Liang, R.; Chen, N.; King, G.; Yu, A.; Chen, Z., Linker-compensated metal–organic framework with electron delocalized metal sites for bifunctional oxygen electrocatalysis. *JACS* **2022,** 144, (11), 4783-4791.
8. Janani, G.; Surendran, S.; Choi, H.; Han, M. K.; Sim, U., In Situ Grown CoMn2O4 3D‐Tetragons on Carbon Cloth: Flexible Electrodes for Efficient Rechargeable Zinc–Air Battery Powered Water Splitting Systems. *Small* **2021,** 17, (47), 2103613.
9. Go, Y.; Min, K.; An, H.; Kim, K.; Shim, S. E.; Baeck, S.-H., Oxygen-vacancy-rich CoFe/CoFe2O4 embedded in N-doped hollow carbon spheres as a highly efficient bifunctional electrocatalyst for Zn–air batteries. *Chem. Eng. J.* **2022,** 448, 137665.
10. Yan, L.; Xu, Z.; Hu, W.; Ning, J.; Zhong, Y.; Hu, Y., Formation of sandwiched leaf-like CNTs-Co/ZnCo2O4@ NC-CNTs nanohybrids for high-power-density rechargeable Zn-air batteries. *Nano Energy* **2021,** 82, 105710.
11. Hong, J. H.; Park, G. D.; Kang, Y. C., Aerosol‐assisted synthesis of bimetallic nanoparticle‐loaded bamboo‐like N‐doped carbon nanotubes as an efficient bifunctional oxygen catalyst for Zn‐air batteries. *Int. J. Energy Res.* **2022,** 46, (4), 5215-5225.
12. Yan, L.; Wang, H.; Shen, J.; Ning, J.; Zhong, Y.; Hu, Y., Formation of mesoporous Co/CoS/Metal-NC@ S, N-codoped hairy carbon polyhedrons as an efficient trifunctional electrocatalyst for Zn-air batteries and water splitting. *Chem. Eng. J.* **2021,** 403, 126385.
13. Wang, A.; Zhao, C.; Yu, M.; Wang, W., Trifunctional Co nanoparticle confined in defect-rich nitrogen-doped graphene for rechargeable Zn-air battery with a long lifetime. *Appl. Catal., B* **2021,** 281, 119514.

14. Luo, F.; Zhu, J.; Ma, S.; Li, M.; Xu, R.; Zhang, Q.; Yang, Z.; Qu, K.; Cai, W.; Chen, Z., Regulated coordination environment of Ni single atom catalyst toward high-efficiency oxygen electrocatalysis for rechargeable Zinc-air batteries. *Energy Storage Mater.* **2021,** 35, 723-730.
15. Yu, N.-F.; Wu, C.; Huang, W.; Chen, Y.-H.; Ruan, D.-Q.; Bao, K.-L.; Chen, H.; Zhang, Y.; Zhu, Y.; Huang, Q.-H., Highly efficient Co3O4/Co@ NCs bifunctional oxygen electrocatalysts for long life rechargeable Zn-air batteries. *Nano Energy* **2020,** 77, 105200.
16. Lei, Z.; Tan, Y.; Zhang, Z.; Wu, W.; Cheng, N.; Chen, R.; Mu, S.; Sun, X., Defects enriched hollow porous Co-N-doped carbons embedded with ultrafine CoFe/Co nanoparticles as bifunctional oxygen electrocatalyst for rechargeable flexible solid zinc-air batteries. *Nano Res.* **2021,** 14, 868-878.
17. Liu, T.; Mou, J.; Wu, Z.; Lv, C.; Huang, J.; Liu, M., A Facile and Scalable Strategy for Fabrication of Superior Bifunctional Freestanding Air Electrodes for Flexible Zinc–Air Batteries. *Adv. Funct. Mater.* **2020,** 30, (36), 2003407.
18. Yang, G.; Zhu, J.; Yuan, P.; Hu, Y.; Qu, G.; Lu, B.-A.; Xue, X.; Yin, H.; Cheng, W.; Cheng, J., Regulating Fe-spin state by atomically dispersed Mn-N in Fe-NC catalysts with high oxygen reduction activity. *Nat. Commun.* **2021,** 12, (1), 1734.
19. Wang, Z.; Ang, J.; Liu, J.; Ma, X. Y. D.; Kong, J.; Zhang, Y.; Yan, T.; Lu, X., FeNi alloys encapsulated in N-doped CNTs-tangled porous carbon fibers as highly efficient and durable bifunctional oxygen electrocatalyst for rechargeable zinc-air battery. *Appl. Catal., B* **2020,** 263, 118344.
20. Pei, Z.; Yuan, Z.; Wang, C.; Zhao, S.; Fei, J.; Wei, L.; Chen, J.; Wang, C.; Qi, R.; Liu, Z.; Chen, Y., A Flexible Rechargeable Zinc–Air Battery with Excellent Low-Temperature Adaptability. *Angew. Chem. Int. Ed.* **2020,** 59, (12), 4793-4799.
21. Tan, Y.; Zhang, Z.; Lei, Z.; Yu, L.; Wu, W.; Wang, Z.; Cheng, N., Electronic modulation optimizes OH* intermediate adsorption on Co-Nx-C sites via coupling CoNi alloy in hollow carbon nanopolyhedron toward efficient reversible oxygen electrocatalysis. *Appl. Catal., B* **2022,** 304, 121006.
22. Zhu, P.; Gao, J.; Liu, S., Facile in situ coupling CoFe/Co nanoparticles and N-doped carbon nanotubes/graphitic nanosheets as bifunctional oxygen electrocatalysts for rechargeable Zn-air batteries. *J. Power Sources* **2020,** 449, 227512.
23. Kumar, Y.; Kibena‐Põldsepp, E.; Mooste, M.; Kozlova, J.; Kikas, A.; Aruväli, J.; Käärik, M.; Kisand, V.; Leis, J.; Tamm, A., Iron and Nickel Phthalocyanine‐Modified Nanocarbon Materials as Cathode Catalysts for Anion‐Exchange Membrane Fuel Cells and Zinc‐Air Batteries. *ChemElectroChem* **2022,** 9, (20), e202200717.
